# Chronic noncommunicable diseases and absenteeism from work: National Survey of Health, 2019

**DOI:** 10.1590/1980-549720240061

**Published:** 2024-12-16

**Authors:** Bernardo Soares do Amaral Fernandes, Milena Maria Tavares Spezani, Leonardo Côrtes Bosco, Beatriz Quintanilha Paladino Tavares de Souza, Giovanni Hora Viviani, Lara Santana Lima da Cunha, Ana Sara Semeão de Souza

**Affiliations:** I Universidade Estácio de Sá, School of Medicine – Rio de Janeiro (RJ), Brazil.

**Keywords:** Absenteeism, Noncommunicable diseases, Multimorbidity, Cross-sectional studies, Health surveys

## Abstract

**Objective:**

To evaluate the association between burden of disease and multimorbidity and absenteeism in Brazil.

**Methods:**

This is a cross-sectional study using data from the National Survey of Health 2019. The assessed outcome was absenteeism from work. The burden of disease was assessed by simply counting a list of 14 morbidities and multimorbidity was defined as: ≥two chronic diseases. Poisson regression models stratified by sex were used to estimate crude and adjusted prevalence ratios and their respective 95% confidence intervals.

**Results:**

Of the 96,131,029 employed individuals, 38.5% reported absenteeism (95%CI 32.9–44.3). The most prevalent morbidities among women who reported absenteeism were back problems (50.8%), depression (42.9%), and hypertension (41.6%); and among men, hypertension (39.7%), chronic back pain (34.1%), and dyslipidemia (19.9%), among those who reported absenteeism. Having multimorbidity increased the report of absenteeism among women by 73% (95%CI 1.01–2.96); among men, there was no association after progressive adjustment for sociodemographic and health factors [PR 1.27 (95%CI 0.96–1.71)].

**Conclusion:**

The burden of disease and multimorbidity are highly prevalent among employed individuals and are strongly related to absenteeism from work, especially among women. In this sense, workers must be the target of interventions to reduce the impact of chronic noncommunicable diseases.

## INTRODUCTION

With the progressive aging of the population, social and economic changes, the urbanization process, and globalization have impacted the way the population lives, works, and eats, contributing to the increase in chronic noncommunicable diseases (NCDs)^1^. The rapid increase in the number of people living with one or more NCD has affected different sectors, including social and economic aspects related to work^2^.

Sick leave is related to several factors, including the cause of the sick leave, age, sex, and work environment^3^. Given its high cost and negative impact on workers’ quality of life, absenteeism due to illness is a major public health issue^2,3^.

In Brazil, NCDs are the biggest cause of death among the population^4^. In 2019, around 50% of the Brazilian population had at least one diagnosed NCD^5^. Economic growth is estimated to be reduced by up to 0.5% for every 10% increase in mortality due to NCDs, making it a major global threat to economic development^1^.

Between 2015 and 2019, 2,934,155 work accidents or occupational diseases were recorded in Brazil, 34% of which resulted in absence from work for more than 15 days^6^. Some authors of studies on absenteeism due to illness have found that diseases of the circulatory system, mental health problems, musculoskeletal disorders, trauma in different parts of the body, and diseases of the respiratory system, among others, are predictors of absenteeism from work^3,7,8^.

Among workers with NCDs, absenteeism is 6.3 times higher when compared to their peers without NCDs^9^. Furthermore, among those with multimorbidity — two or more NCDs at the same time —, the loss of productivity is on average 170 hours per worker each year, accounting for hours lost due to absenteeism and presenteeism, directly impacting the economy and personal income in the employment relationship^8^.

Despite the extensive loss of productivity at work related to NCDs, this relationship in the Brazilian population is still not well understood. Evidence concerning the influence of health conditions on absenteeism assessed specific groups of workers^7,10–12^. In addition, differences between the sexes have been little explored in the Brazilian context. Thus, knowing the relationship between the burden of disease and multimorbidity in absenteeism can contribute to a better understanding of the impact of NCDs on Brazilian workers, in addition to providing evidence for formulating policies that aim to improve the quality of life of workers with NCDs, partially reducing absenteeism from work. Therefore, we aimed to evaluate the association between burden of disease and multimorbidity and absenteeism in Brazil.

## METHODS

This is a cross-sectional study, using the National Survey of Health (*Pesquisa Nacional de Saúde* – PNS) 2019 as a database, conducted by the Brazilian Ministry of Health and the Brazilian Institute of Geography and Statistics (IBGE), and which has public and unrestricted access. The geographic scope is the entire national territory, with the target population being residents of permanent private residencies. Its sampling plan was carried out by three-stage sampling^13^. The population of this study consisted of individuals over 18 years of age employed in the reference week, comprising 94,114 individuals.

According to IBGE, people classified as employed in the reference week of the survey are those who, in that period: worked at least one full hour in jobs paid in money, products, goods, or benefits (housing, food, clothing, training, etc.) or in jobs without direct pay to help with the economic activity of a household member or, even, people who had paid jobs from which they were temporarily absent during that week.

The outcome of this study was absenteeism measured through self-report (yes/no), through the question: "*In the reference week, did you have any paid or unpaid work from which you were temporarily absent*?". Cases of absence due to vacation, maternity leave, and occasional factors, such as bad weather and interruptions in transport services, were excluded.

The exposure variables evaluated were the burden of disease and multimorbidity, based on a list of 14 NCDs, available in the PNS, whether they were work-related or not. Therefore, the following NCDs were evaluated: cerebrovascular accident (CVA), asthma, cancer, diabetes mellitus, work-related musculoskeletal disorders (WMSDs), chronic obstructive pulmonary disease (COPD), depression, dyslipidemia, cardiovascular diseases (CVD) — acute myocardial infarction and unstable angina —, systemic arterial hypertension (SAH), chronic kidney disease (CKD), chronic back pain, mental health problems, and rheumatism. Self-reporting of chronic conditions was obtained by asking "*Has a doctor ever diagnosed you with…*".

The burden of disease was assessed by simply counting morbidities, each with a weight equal to one. Multimorbidity was assessed as the presence of two or more self-reported morbidities in the same individual^14^.

The independent variables evaluated were: sex (men/women), age categorized in years (18 to 29, 30 to 49, 40 to 49, 50 to 59, 60 years or older), level of education (illiterate, elementary school, high school, and college degree), self-declared skin color/ethnicity (white, Black, brown), categorized per capita income (<1 minimum wage, 1 to 3, >3 minimum wages), health insurance plan (yes/no), self-rated health status (very good, good, fair, poor, very poor).

The prevalence (%) of each of the chronic diseases was estimated for the burden of disease and multimorbidity according to absenteeism stratified by sex. Crude and adjusted prevalence ratios (PR) were also calculated between absenteeism, burden of disease, and multimorbidity, overall and stratified by sex. The crude models (model 1) were progressively (stepwise) adjusted for sociodemographic (model 2: age, level of education, and ethnicity/skin color), and health (model 3: self-rated health) factors. Residual analysis was performed to verify the adequacy of the models. Prevalence ratios and their respective 95% confidence intervals (95%CI) were estimated using Poisson regression models.

The analyses were performed using the Stata SE 15.0 software, using the "svy" command, considering the weights of the individuals and the sampling parameters in all analyses, as surveys that use complex sampling present different probabilities of selecting clusters and individuals.

The PNS 2019 was approved by the National Commission of Ethics in Research — CONEP (*Comissão Nacional de Ética em Pesquisa*), of the National Health Council — CNS (*Conselho Nacional de Saúde*), in August 2019. All participants signed an Informed Consent Form before the interviews began. Furthermore, all regulatory and legal aspects were complied with. The data are publicly accessible and the identity of the subjects is preserved. For the present research, the database was extracted and analyzed in November 2023.

## RESULTS

A total of 96,131,029 people participated in the study, the majority of whom were men (55.5%), aged 30 to 39 years (27.3%), holding a high school degree (39.5%), white skin color/ethnicity (44.7%), per capita income of up to one minimum wage (43.9%), and without health insurance coverage (69.8%). The most commonly self-rated health status was "good" (56.8%). The prevalence of absenteeism was 38.5%; about 50% of the population reported not having any of the assessed NCDs and the prevalence of multimorbidity was 23.6% (Table 1).

**Table 1:** Demographic, socioeconomic, and health characteristics of the study population. Brazil, 2019.

Variables		n	%	95%CI
Sex				
	Men	53,337,106	55.5	(54.7–56.3)
	Women	42,793,923	44.5	(43.7–45.4)
Age				
	18 to 29	22,248,733	23.2	(22.3–24.0)
	30 to 39	25,954,099	27.0	(26.3–27.7)
	40 to 49	22,001,675	22.9	(22.3–23.6)
	50 to 59	17,531,087	18.2	(17.7–18.8)
	60 years or older	7,229,427	8.7	(8.3–9.2)
Level of education				
	Illiterate	2,634,465	2.7	(2.5–3.0)
	Elementary school	36,062,704	37.5	(36.7–38.4)
	High school	37,960,994	39.5	(38.7–40.3)
	College degree	19,472,866	20.3	(19.4–21.1)
Ethnicity/skin color				
	White	42,982,040	44.7	(43.8–45.6)
	Black	11,593,577	12.1	(11.6–12.6)
	Brown	41,544,891	43.2	(42.4–44.1)
Per capita income				
	Up to 1 wage	42,239,193	44.0	(43.0–44.9)
	1 to 3 wages	40,857,262	42.5	(41.6–43.4)
	3+ wages	12,989,641	13.5	(12.8–14.3)
Health insurance plan				
	Yes	29,076,755	30.3	(29.3–31.2)
	No	67,054,274	69.8	(68.8–70.7)
Self-rated health				
	Very good	18,070,707	18.8	(18.1–19.5)
	Good	54,551,561	56.8	(55.9–57.6)
	Fair	20,864,014	21.7	(21.0–22.4)
	Poor	2,231,850	2.3	(2.1–2.5)
	Very poor	412,898	0.4	(0.4–0.5)
Absenteeism				
	Yes	852,642	38.5	(32.9–44.3)
	No	1,362,988	61.5	(55.7–67.1)
Morbidities				
	0	43,107,450	49.9	(49.0–50.8)
	1	22,916,220	26.5	(25.8–27.2)
	2	11,192,362	12.9	(12.4–13.5)
	3	5,200,828	6.0	(5.6–6.4)
	4	2,356,031	2.7	(2.5–3.0)
	5	1,039,829	1.2	(1.0–1.4)
	6+	652.340	0.8	(0.6–0.9)
Multimorbidity				
	Yes	20,441,390	23.6	(22.9–24.4)
	No	66,023,671	76.4	(75.6–77.1)

n: absolute number; %: proportion; 95%CI: 95% confidence interval.

**Figure 1:**
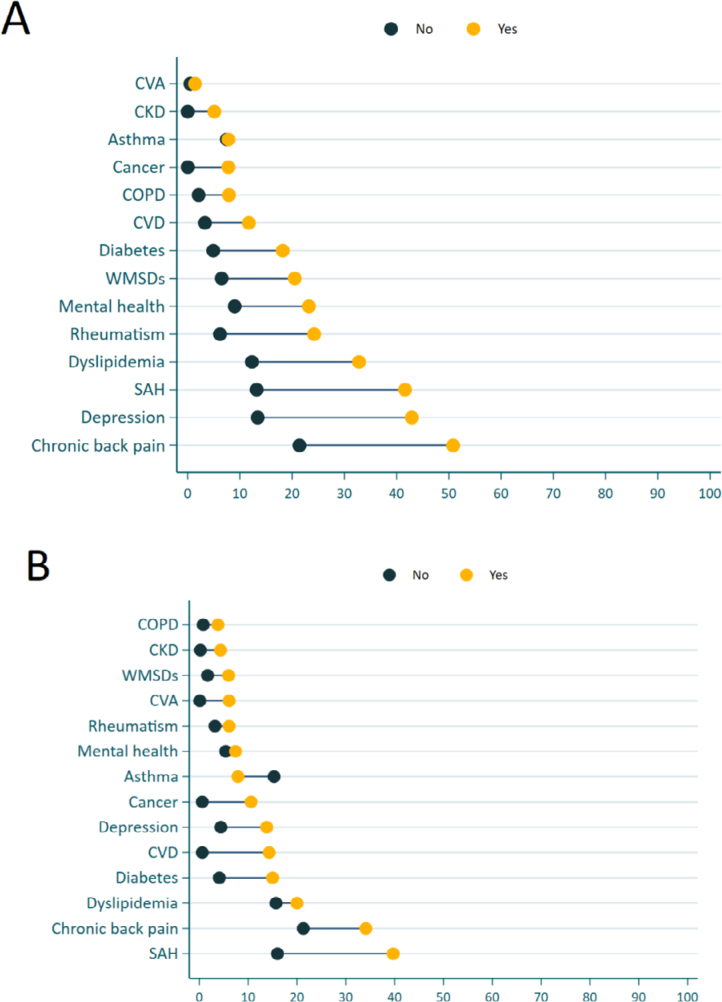
Prevalence of chronic noncommunicable diseases according to absenteeism stratified by sex: (A) women and (B) men. Brazil, 2019.

CVA: cerebrovascular accident; WMSDs: work-related musculoskeletal disorders; COPD: chronic obstructive pulmonary disease; CVD: cardiovascular diseases; SAH: systemic arterial hypertension; CKD: chronic kidney disease.

In Figure 1 we show the prevalence of NCDs according to absenteeism stratified by sex. Men and women who reported absenteeism showed a similar pattern of more prevalent NCDs; however, the prevalence was higher in women compared to men. The most prevalent NCDs among women who reported absenteeism were: back problems (50.8%), depression (42.9%), and SAH (41.6%) (Figure 1A). Conversely, among men who reported absenteeism, the most common NCDs were SAH (39.7%), chronic back pain (34.1%), and dyslipidemia (19.9%) (Figure 1B).

**Figure 2:**
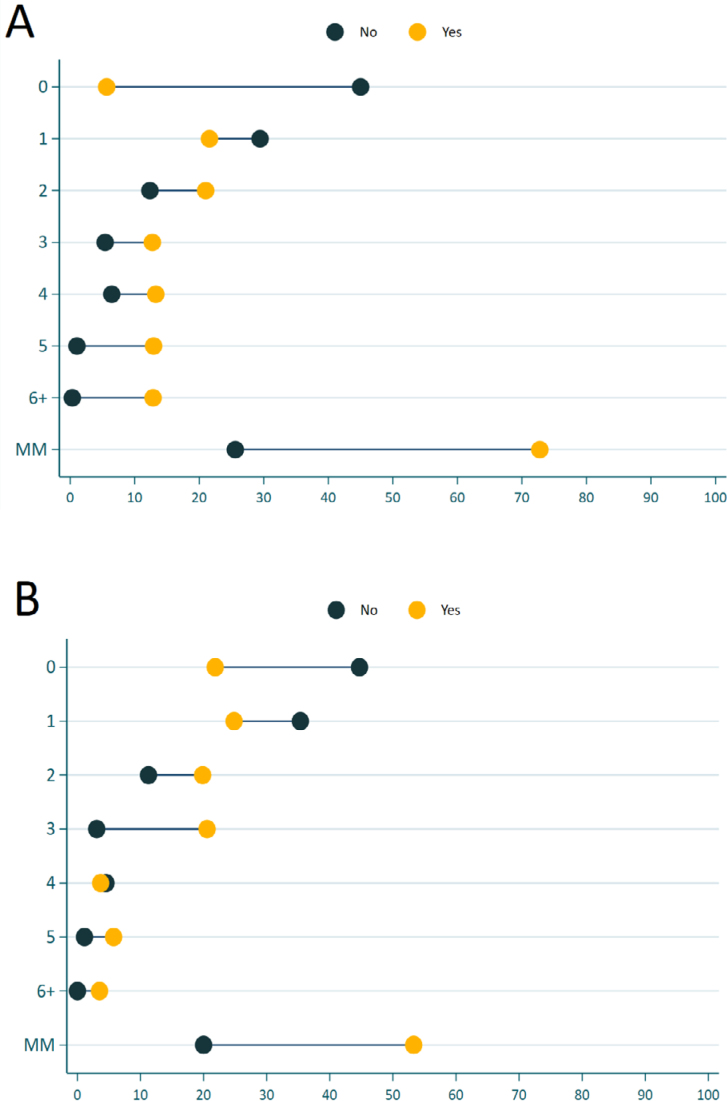
Prevalence of absenteeism according to burden of disease stratified by sex: (A) women and (B) men. Brazil, 2019.

In the analysis of the prevalence of absenteeism in relation to the burden of disease, men and women who did not report absenteeism had higher prevalence for a smaller number of NCDs (0 and 1). From two morbidities on, the prevalence is higher among people who reported absenteeism (Figure 2). Nonetheless, for men with absenteeism from four morbidities on, the prevalence is lower compared to women with absenteeism, and we also observed a reduction in the differences in prevalence compared to men who did not report absenteeism (Figure 2B).

MM: multimorbidity.

In Table 2 we present the crude and adjusted prevalence ratios for absenteeism, burden of disease, and multimorbidity for the general population and stratified by sex. In the general population, having multimorbidity increased the report of absenteeism from work by 2.6 times compared to individuals without multimorbidity. After progressive adjustment of the models for sociodemographic and health factors, there was a reduction in the strength of association. Overall, having multimorbidity increased the reported absenteeism by 38% (95%CI 1.04–1.82). Regarding the burden of disease, even after progressive adjustment, the increase in the number of morbidities raised the reporting of absenteeism, being 60% higher (95%CI 1.02–2.52) among those with two morbidities and 2.23 times higher (95%CI 1.49–3.35) among those with six or more morbidities (Table 2).

**Table 2:** Crude and adjusted prevalence ratios (PR) of the association between multimorbidity and burden of disease with absenteeism, overall and stratified by sex.

Variables		Overall			Women			Men		
		Model 1 PR (95%CI)	Model 2 PR (95%CI)	Model 3 PR (95%CI)	Model 1 PR (95%CI)	Model 2 PR (95%CI)	Model 3 PR (95%CI)	Model 1 PR (95%CI)	Model 2 PR (95%CI)	Model 3 PR (95%CI)
Multimorbidity		2.60 (1.96–3.45)	1.72 (1.32–2.26)	1.38 (1.04–1.82)	4.11 (2.45–6.88)	2.52 (1.49–4.26)	1.73 (1.01–2.96)	1.94 (1.39–2.70)	1.43 (1.10–1.87)	1.27 (0.96–1.71)
Burden of disease										
	1	1.87 (1.17–2.96)	1.64 (1.08–2.50)	1.49 (0.99–2.24)	4.71 (2.10–10.58)	4.00 (1.85–8.61)	3.14 (1.50–6.58)	1.25 (0.77–2.03)	1.18 (0.76–1.74)	1.16 (0.75–1.79)
	2	3.02 (1.94–4.70)	2.05 (1.34–3.15)	1.60 (1.02–2.52)	8.36 (4.09–17.08)	5.6 (2.69–11.71)	3.54 (1.62–7.78)	1.91 (1.17–3.11)	1.45 (0.95–2.21)	1.29 (0.80–2.08)
	3	4.10 (2.72–6.16)	2.60 (1.70–3.97)	2.00 (1.31–3.05)	10.0 (4.94–20.23)	7.05 (3.25–15.29)	4.56 (2.21–9.43)	2.57 (1.67–3.97)	1.74 (1.18–2.56)	1.52 (1.01–2.28)
	4	2.70 (1.54–4.74)	2.01 (1.22–3.30)	1.28 (0.78–2.12)	9.32 (4.19–20.74)	6.28 (2.80–14.06)	2.46 (1.02–5.96)	1.36 (0.61–3.06)	1.17 (0.64–2.13)	1.11 (0.62–1.97)
	5	4.92 (3.23–7.50)	2.59 (1.56–4.29)	2.13 (1.30–3.49)	16.95 (8.66–33.16)	7.4 (3.43–16.03)	6.25 (3.06–12.76)	2.48 (1.54–4.00)	1.96 (1.00–3.82)	1.53 (0.81–2.91)
	6+	5.61 (3.8–8.26)	3.04 (1.98–4.66)	2.23 (1.49–3.35)	19.07 (10.13–35.88)	9.17 (4.57–18.39)	4.76 (2.33–9.77)	2.94 (1.94–4.44)	1.67 (1.17–2.40)	1.58 (1.11–2.26)

95%CI: 95% confidence interval. Model 1: crude model; Model 2: age, level of education, and ethnicity/skin color; Model 3: model 2 + self-rated health.

In the analysis stratified by sex, having multimorbidity increased the report of absenteeism among women by 73% (95%CI 1.01–2.96); among men, there was no association after progressive adjustment for sociodemographic and health factors [PR 1.27 (95%CI 0.96–1.71)] (Table 2). Among women, the burden of disease was strongly associated with the report of absenteeism, being three times higher (95%CI 1.50–6.58) for those with one morbidity and 6.3 times (95%CI 3.06–12.76) among women with six or more morbidities (Table 2). For men, after progressive adjustment, the association between burden of disease and absenteeism showed statistical significance only among those with six or more morbidities, the reported absenteeism being 58% (95%CI 1.11–2.26) higher among them compared to those without any morbidity (Table 2).

## DISCUSSION

The results of this study evidenced the magnitude of NCDs among employed individuals in Brazil, where about 50% have at least one assessed NCD. Furthermore, the pattern of most prevalent morbidities among men and women who reported absenteeism was different. A quarter of the population has multimorbidity and its prevalence among women who reported absenteeism was substantially higher compared to men. We found significant associations between the number of chronic conditions, multimorbidity, and absenteeism from work, with this relationship being more significant among women.

It is estimated that over 43 million employed Brazilians have at least one assessed NCD, and around 850 thousand reported having missed work in 2019. According to the results of a European study, there was an increase from 3.6 to 5.2 million people who were absent from work due to illness between 2006 and 2020^15^. The prevalence of absenteeism was 38.5% in our sample. Authors of studies conducted in Canada and the United States of America in 2016 found a prevalence of absenteeism of 27.8% and 53% among workers, respectively^16,17^.

Regarding the prevalence of the number of chronic conditions in our study population, Zhang et al. found similar prevalence values for burden of disease, with approximately 45% of the population having at least one chronic disease^16^. As for multimorbidity, we found a prevalence of 23.6%, a higher value than that found in another study whose authors evaluated workers at older ages^18^. Conversely, the prevalence found in our study is almost half when compared to a study carried out with workers in Australia (2020), whose authors found a 53.1% prevalence of multimorbidity for the total population, 63.5% for women and 41.5% for men^8^. It is worth highlighting that the differences found in the prevalence of NCDs in our study and in research carried out in European countries can be partially explained by the epidemiological transition of these countries, which are in more advanced stages, representing the greatest cause of morbidity and mortality in this population, compared to Brazil^19,20^.

The most prevalent diseases in both sexes, among those who reported absenteeism, were chronic back pain, systemic arterial hypertension, and dyslipidemia. The most prevalent chronic conditions, according to some researchers, are: diseases of the musculoskeletal system, mental and behavioral disorders, diseases of the circulatory system, migraines, high cholesterol, diseases of the respiratory system, and diseases of the digestive system^3,8,16,17^.

However, those who evaluated the differences between the sexes found differences in the most prevalent diagnoses between them, as was found in the present research. In the study by Timp et al., the most prevalent diagnoses for women were mental disorders, while for men diseases of the musculoskeletal system were more prevalent^3^.

Regarding absences due to health reasons among women, there is a tendency toward more frequent absences due to mental disorders and musculoskeletal problems such as chronic back pain and diseases of the circulatory system. These patterns may reflect the multiple social roles played by women and gender segregation in the labor market, with this group being more likely to be subjected to occupations characterized by high physical and emotional demands, low wages, limited social support, and unpromising career prospects, increasing the risk of sick leaves^10^.

In addition, women are predominantly employed in the health, social services, and education sectors, while men are more commonly found in jobs in the construction, manufacturing, information technology, and transportation sectors^21^. Some researchers suggest that higher rates of absenteeism due to illness are associated with female-dominated occupations. The emotionally demanding nature of these jobs often involves working directly with patients or clients, requiring a more complete recovery from mental disorders before work can be resumed, for example^21,22^.

Moreover, gender inequality in absenteeism becomes evident when analyzing the results of the study conducted by Almeida and Fernandes, in which the workforce is predominantly male, but absences due to health reasons are more frequent among women. Musculoskeletal diseases, such as problems in the spine, lower back, and shoulders, are responsible for the highest number of sick leaves and lost days, correlated with lower levels of education and roles occupied by women in businesses^23^.

The prevalence of absenteeism among women decreased as of three conditions and remained stable in the subsequent categories. For men, this pattern occurs from four conditions on. Authors of an Australian cohort study on absenteeism among young workers highlight the importance of disease synergy, suggesting a partially additive, rather than multiplicative, effect on the relationship between absenteeism and more severe conditions^8^. According to the authors, the synergistic effects of various disease combinations are likely to range from amplifying the effect of individual conditions to enhancing the effect. Nevertheless, they emphasize that these synergistic effects should be further investigated to understand the combinations of diseases that may be particularly harmful^8^.

Furthermore, according to our results, there is gender inequality in the relationship between burden of disease, multimorbidity, and prevalence of absenteeism. Authors of a study carried out in 2021 analyzed the causes of absenteeism in 32 European countries and found differences in absenteeism rates between men and women. The difference was greater in France, followed by the United Kingdom, Spain, and Poland respectively. In these countries, the same trend was observed: there are higher rates of sick leave in women than in men^15^.

Bekker et al. conducted a literature review on the relationship between gender and sick leave, finding that women are generally absent more frequently, especially when it comes to short-term absences^24^. In addition, they found that gender differences in sick leaves are influenced by several factors such as country of residence, age, and professional group^24^.

Differences in daily activities and occupational characteristics may also influence the frequency and duration of sick leaves. Women generally tend to spend more time on household chores and childcare than men. The double burden hypothesis suggests that combining different roles, such as childcare and working outside the home, can increase stress and, consequently, the risk of sick leave^3,25,26^.

Finally, having multimorbidity was associated with reported absenteeism from work, being more significant among women. Despite differences in the prevalence of multimorbidity according to sex, in the general population, Troelstra et al. found an association between multimorbidity and loss of productivity at work, even when adjusted for occupation^8^. Researchers of another study also demonstrated that people with multimorbidity had higher rates of absenteeism and presenteeism than those with any chronic disease alone or no chronic disease^9^.

Workers with multimorbidity account for a considerable portion of personal expenses and those of the Brazilian Unified Health System (SUS) due to their illnesses. In Brazil, in 2013, there were 974,641 hospital admissions due to NCDs, totaling a cost of BRL 1,848,627,410.03 (USD 695.6 million) for the SUS^27^. An alternative to reducing the impact of NCDs on workers’ health is to understand that the workplace has a direct influence on their health and lifestyle, as they spend a considerable amount of time in this environment^28^.

By identifying the most common illnesses that lead to absenteeism, it is possible to develop occupational health policies focused on the prevention and treatment of these conditions. This may include vaccination programs, awareness campaigns, regular checkups, workplace ergonomics, and emotional support.

Furthermore, understanding the magnitude and impact of NCDs can help in the formulation of more flexible work policies such as home office or reduced working hours. These alternatives can allow workers to continue contributing without the need for extended leaves. In addition, supporting workers’ mental and physical health creates a healthier, more collaborative environment.

Strengths of the present study include the use of nationally representative data. The use of periodic data, such as the PNS, allows future analyses regarding the behavior and monitoring of the study population. Limitations of the study include the use of self-reported measures of morbidity and absenteeism that may underestimate their prevalence. Furthermore, all assessed diseases were considered equally, although the effect of burden of disease and multimorbidity may vary with the combination and severity of NCDs^14^.

It should be noted that the set of 14 chronic conditions evaluated may have underestimated the estimated prevalence of burden of disease and multimorbidity in the study population. Moreover, the variability in the number and list of diseases included in previous studies makes it difficult to compare prevalence and its impact on absenteeism. Another important limitation is that absenteeism was assessed as absence (yes/no) from work, without considering the time away. Therefore, the results should be interpreted considering that the relationship between NCDs and loss of productivity may be different from that found by the report measured dichotomously. In addition, as this was a cross-sectional study, the associations found here did not assess causality, considering that exposure and outcome were simultaneously assessed.

All in all, we conclude that NCDs are already highly prevalent among Brazilian workers and that the burden of disease and multimorbidity are strongly related to absenteeism from work, especially among women. Considering that the increase in NCDs in the coming years and in the dependency ratio, with an aging population, will place a greater burden on young workers, combating the effects of NCDs in this population is essential for their employers and for society in general.

Finally, these results highlight the importance of prevention and early management aimed at minimizing the impact of chronic noncommunicable diseases among workers. Among the targeted actions are: strategies to reduce multimorbidity, increasing workers’ ability to deal with their conditions, and improving access to health services for prevention and treatment for workers with illnesses, especially women.
