# Supplementary material for: Chronic noncommunicable diseases and absenteeism from work: National Survey of Health, 2019
Source: Rev Bras Epidemiol. 2024 Dec 16;27:e240061. doi: 10.1590/1980-549720240061 (PMC11654286; doi:10.1590/1980-549720240061)
Supplement: Translation

# Translation: Doenças crônicas não transmissíveis e absenteísmo no trabalho: Pesquisa Nacional de Saúde 2019

**DOI:** 10.1590/1980-549720240061.2

**Authors:** Bernardo Soares do Amaral Fernandes, Milena Maria Tavares Spezani, Leonardo Côrtes Bosco, Beatriz Quintanilha Paladino Tavares de Souza, Giovanni Hora Viviani, Lara Santana Lima da Cunha, Ana Sara Semeão de Souza

**Affiliations:** I Universidade Estácio de Sá, Faculdade de Medicina – Rio de Janeiro (RJ), Brasil.

**Keywords:** Absenteísmo, Doenças não transmissíveis, Multimorbidade, Estudos transversais, Inquéritos epidemiológicos

## Abstract

**Objetivo:**

Avaliar a associação entre carga de morbidade e multimorbidade e absenteísmo no Brasil.

**Método:**

Estudo transversal utilizando dados da Pesquisa Nacional de Saúde 2019. O desfecho avaliado foi absenteísmo no trabalho. Carga de morbidade foi avaliada pela contagem simples de uma lista de 14 morbidades e multimorbidade foi definida como: ≥duas doenças crônicas. Modelos de regressão de *Poisson* estratificados por sexo foram utilizados para estimar as razões de prevalência brutas e ajustadas e seus respectivos intervalos de confiança de 95%

**Resultado:**

Dos 96.131.029 indivíduos em condição de ocupação, 38,5% relataram absenteísmo (IC95% 32,9–44,3). As morbidades mais prevalentes entre as mulheres que relataram absenteísmo foram: problemas de coluna (50,8%), depressão (42,9%) e hipertensão (41,6%); e entre os homens foram: hipertensão (39,7%), problema crônico de coluna (34,1%) e dislipidemia (19,9%), entre aqueles que relataram absenteísmo. Ter multimorbidade aumentou 73% o relato de absenteísmo na população feminina (IC95% 1,01–2,96), entre os homens não houve associação após ajuste progressivo por fatores sociodemográficos e de saúde [RP 1,27 (IC95% 0,96–1,71)].

**Conclusão:**

A carga de morbidade e a multimorbidade são altamente prevalentes entre indivíduos em condição de ocupação e está fortemente relacionada com o absenteísmo no trabalho, especialmente entre as mulheres. Nesse sentido, trabalhadores devem ser alvo de intervenções que visem reduzir o impacto das condições crônicas não transmissíveis entre eles.

## INTRODUÇÃO

Com o progressivo envelhecimento da população, as mudanças sociais e econômicas, o processo de urbanização e a globalização impactaram o modo de viver, de trabalhar e de se alimentar da população, contribuindo para o aumento das doenças crônicas não transmissíveis (DCNT)^1^. O rápido aumento no número de pessoas que vivem com uma ou mais DCNT tem afetado diferentes setores, incluindo os aspectos sociais e econômicos relacionados ao trabalho^2^.

O afastamento por doença está relacionado a vários fatores, incluindo a causa desse afastamento, a idade, o sexo e o ambiente de trabalho^3^. Dado o seu elevado custo e o impacto negativo na qualidade de vida dos trabalhadores, o absenteísmo por doença é um importante problema de saúde pública^2,3^.

No Brasil, as DCNT são a maior causa de morte da população^4^. Em 2019, cerca de 50% da população brasileira possuía ao menos uma DCNT diagnosticada^5^. Estima-se que o crescimento econômico seja reduzido em até 0,5% para cada incremento de 10% na mortalidade por DCNT, tornando-se uma importante ameaça global ao desenvolvimento econômico^1^.

Entre 2015 e 2019, foram registrados 2.934.155 acidentes de trabalho ou doenças ocupacionais no Brasil, sendo que 34% destes resultaram em um afastamento do trabalho por mais de 15 dias^6^. Alguns estudos sobre absenteísmo por doença encontraram como preditores da ausência no trabalho doenças do aparelho circulatório, problemas de saúde mental, distúrbios musculoesqueléticos, traumas em diferentes partes do corpo e doenças do aparelho respiratório, entre outros^3,7,8^.

Entre trabalhadores com DCNT, o absenteísmo é 6,3 vezes maior quando comparado aos seus pares sem DCNT^9^. Ademais, entre aqueles com multimorbidade — duas ou mais DCNT ao mesmo tempo —, a perda de produtividade é em média de 170 horas por trabalhador a cada ano, contabilizando horas perdidas devido ao absenteísmo e ao presenteísmo, impactando diretamente a economia e o rendimento pessoal no vínculo empregatício^8^.

Apesar da extensa perda de produtividade no trabalho relacionada às DCNT, essa relação na população brasileira ainda não é bem compreendida. As evidências que avaliaram a influência das condições de saúde no absenteísmo avaliaram grupos de trabalhadores específicos^7,10–12^. Além disso, as diferenças entre os sexos têm sido pouco exploradas no contexto brasileiro. Dessa forma, conhecer sobre a relação entre a carga de morbidade e de multimorbidade no absenteísmo pode contribuir para uma melhor compreensão sobre o impacto das DCNT em pessoas em condição de ocupação no Brasil; fornecendo evidências para a construção de políticas que visem melhorar a qualidade de vida dos trabalhadores com DCNT, reduzindo em parte, o absenteísmo no trabalho. Portanto, o objetivo deste estudo foi avaliar a associação entre carga de morbidade e de multimorbidade e absenteísmo no Brasil.

## MÉTODOS

Trata-se de um estudo transversal, utilizando como banco de dados a Pesquisa Nacional de Saúde (PNS): 2019, conduzida pelo Ministério da Saúde e pelo Instituto Brasileiro de Geografia e Estatística (IBGE), e que possui acesso público e irrestrito. A abrangência geográfica é todo o território nacional, tendo como população-alvo os moradores de domicílios particulares permanentes. Seu plano amostral foi realizado por amostragem em três estágios^13^. A população deste estudo foi constituída por indivíduos maiores de 18 anos em condição de ocupação na semana de referência, composta por 94.114 indivíduos.

Segundo o IBGE, são classificadas como ocupadas na semana de referência da pesquisa as pessoas que, nesse período: trabalharam pelo menos uma hora completa em trabalho remunerado em dinheiro, produtos, mercadorias ou benefícios (moradia, alimentação, roupas, treinamento, etc.) ou em trabalho sem remuneração direta em ajuda à atividade econômica de membro do domicílio, ou, ainda, as pessoas que tinham trabalho remunerado do qual estavam temporariamente afastadas nessa semana.

O desfecho deste estudo foi o absenteísmo medido através do autorrelato (sim/não), por meio do questionamento: "*Na semana de referência, tinha algum trabalho remunerado ou não do qual estava temporariamente afastado*". Foram excluídos os casos de afastamentos por férias, licença maternidade e fatores ocasionais como mau tempo e paralisação nos serviços de transporte.

As variáveis de exposição avaliadas foram a carga de morbidade e de multimorbidade, baseado em uma lista de quatorze DCNT, disponíveis na PNS, sendo elas relacionadas ao trabalho ou não. Por conseguinte, foram avaliadas: acidente vascular cerebral (AVC), asma, câncer, diabetes mellitus, distúrbios osteomusculares relacionados ao trabalho (DORT), doença pulmonar obstrutiva crônica (DPOC), depressão, dislipidemia, doenças cardiovasculares (DCV) — infarto agudo do miocárdio e angina instável —, hipertensão arterial sistêmica (HAS), insuficiência renal crônica (IRC), problema crônico de coluna, problemas de saúde mental e reumatismo. O autorrelato de condições crônicas foi obtido por meio do questionamento "*Algum médico já lhe deu o diagnóstico de…*".

A carga de morbidade foi avaliada pela contagem simples de morbidades, tendo cada uma delas peso um. A multimorbidade foi avaliada como a presença de duas ou mais morbidades autorrelatas em um mesmo indivíduo^14^.

As variáveis independentes avaliadas foram: sexo (masculino/feminino), idade categorizada em anos (18 a 29, 30 a 49, 40 a 49, 50 a 59, 60 anos ou mais), nível de instrução (sem instrução, fundamental completo, ensino médio completo e ensino superior completo), cor/raça autodeclarada (branca, preta, parda), renda *per capita* categorizada (<1 salário mínimo, de 1 a 3, >3 salários mínimos), plano de saúde (sim/não), autoavaliação do estado de saúde (muito bom, bom, regular, ruim, muito ruim).

Foram calculadas as prevalências (%) de cada uma das doenças crônicas para a carga de morbidade e de multimorbidade segundo absenteísmo estratificado por sexo. Também foram calculadas as razões de prevalência (RP) bruta e ajustada entre o absenteísmo, carga de morbidade e de multimorbidade, geral e estratificado por sexo. Os modelos brutos (modelo 1) foram progressivamente (*stepwise*) ajustados por fatores sociodemográficos (modelo 2: idade, escolaridade e raça/cor) e de saúde (modelo 3: autoavaliação de saúde). Foi realizada a análise de resíduo para verificar a adequação dos modelos. As razões de prevalências e seus respectivos intervalos de confiança a 95% (IC95%) foram estimadas por meio de modelos de regressão de *Poisson*.

As análises foram realizadas no *software* "Stata SE 15.0", por meio do comando "svy", considerando os pesos dos indivíduos e os parâmetros amostrais em todas as análises, visto que os inquéritos que utilizam amostragem complexa apresentam probabilidades de seleção de conglomerados e indivíduos diferentes.

A PNS 2019 foi aprovada pela Comissão Nacional de Ética em Pesquisa — CONEP, do Conselho Nacional de Saúde — CNS, em agosto de 2019. Todos os participantes assinaram um Termo de Consentimento Livre e Esclarecido antes do início das entrevistas. Outrossim, todos os aspectos regulatórios e legais foram cumpridos. Os dados são de acesso público e preservam a identidade dos sujeitos. Para a presente pesquisa, o banco de dados foi extraído e analisado em novembro de 2023.

## RESULTADOS

Fizeram parte do estudo 96.131.029 pessoas, sendo a maioria homens (55,5%), de 30 a 39 anos (27,3%), com ensino médio completo (39,5%), cor/raça branca (44,7%), renda *per capita* de até 1 salário-mínimo (43,9%) e sem cobertura de plano de saúde (69,8%). O estado de saúde mais comumente autorrelatado foi "bom" (56,8%). A prevalência de absenteísmo foi de 38,5%; cerca de 50% da população relatou não ter nenhuma DCNT avaliada e a prevalência de multimorbidade foi de 23,6% (Tabela 1).

**Tabela 1:** Características demográficas, socioeconômicas e de saúde da população de estudo. Brasil, 2019.

Variáveis		n	%	IC95%
Sexo				
	Masculino	53.337.106	55,5	(54,7–56,3)
	Feminino	42.793.923	44,5	(43,7–45,4)
Idade				
	18 a 29	22.248.733	23,2	(22,3–24,0)
	30 a 39	25.954.099	27,0	(26,3–27,7)
	40 a 49	22.001.675	22,9	(22,3–23,6)
	50 a 59	17.531.087	18,2	(17,7–18,8)
	60 anos ou mais	7.229.427	8,7	(8,3–9,2)
Escolaridade				
	Sem instrução	2.634.465	2,7	(2,5–3,0)
	Ens. Fundamental	36.062.704	37,5	(36,7–38,4)
	Ens. Médio	37.960.994	39,5	(38,7–40,3)
	Ens. Superior	19.472.866	20,3	(19,4–21,1)
Raça/Cor				
	Branca	42.982.040	44,7	(43,8–45,6)
	Preta	11.593.577	12,1	(11,6–12,6)
	Parda	41.544.891	43,2	(42,4–44,1)
Renda per capita				
	Até 1 salário	42.239.193	44,0	(43,0–44,9)
	1 até 3 salários	40.857.262	42,5	(41,6–43,4)
	3+ salários	12.989.641	13,5	(12,8–14,3)
Plano de saúde				
	Sim	29.076.755	30,3	(29,3–31,2)
	Não	67.054.274	69,8	(68,8–70,7)
Autoavaliação de saúde				
	Muito bom	18.070.707	18,8	(18,1–19,5)
	Bom	54.551.561	56,8	(55,9–57,6)
	Regular	20.864.014	21,7	(21,0–22,4)
	Ruim	2.231.850	2,3	(2,1–2,5)
	Muito ruim	412.898	0,4	(0,4–0,5)
Absenteísmo				
	Sim	852.642	38,5	(32,9–44,3)
	Não	1.362.988	61,5	(55,7–67,1)
Morbidades				
	0	43.107.450	49,9	(49,0–50,8)
	1	22.916.220	26,5	(25,8–27,2)
	2	11.192.362	12,9	(12,4–13,5)
	3	5.200.828	6,0	(5,6–6,4)
	4	2.356.031	2,7	(2,5–3,0)
	5	1.039.829	1,2	(1,0–1,4)
	6+	652.340	0,8	(0,6–0,9)
Multimorbidade				
	Sim	20.441.390	23,6	(22,9–24,4)
	Não	66.023.671	76,4	(75,6–77,1)

n: número absoluto; %: proporção; IC95%: intervalo de confiança a 95%.

A Figura 1 apresenta a prevalência de DCNT segundo absenteísmo estratificado por sexo. Homens e mulheres que relataram absenteísmo apresentaram um padrão similar de DCNT mais prevalentes, contudo, nas mulheres a prevalência foi maior quando comparada aos homens. As DCNT mais prevalentes entre as mulheres que relataram absenteísmo foram: problemas de coluna (50,8%), depressão (42,9%) e HAS (41,6%) (Figura 1A). Já entre os homens, as DCNT mais presentes foram HAS (39,7%), problema crônico de coluna (34,1%) e dislipidemia (19,9%), entre aqueles que relataram absenteísmo (Figura 1B).

**Figura 1:** Prevalência de doenças crônicas não transmissíveis segundo absenteísmo estratificado por sexo: (A) mulheres e (B) homens. Brasil, 2019.

AVC: acidente vascular cerebral; DORT: distúrbios osteo musculares relacionados ao trabalho; DPOC: doença pulmonar obstrutiva crônica; DCV: doenças cardiovasculares; HAS: hipertensão arterial sistêmica; IRC: insuficiência renal crônica.

Na análise da prevalência de absenteísmo em relação à carga de morbidade, homens e mulheres que não relataram absenteísmo apresentam prevalências mais altas para um número menor de DCNT (0 e 1). A partir de duas morbidades, a prevalência é maior entre as pessoas que relataram absenteísmo (Figura 2). Entretanto, para os homens com absenteísmo a partir de quatro morbidades, a prevalência é menor em comparação às mulheres com absenteísmo, e também é possível observar uma redução das diferenças na prevalência em comparação aos homens que não relataram absenteísmo (Figura 2B).

**Figura 2:** Prevalência de absenteísmo segundo carga de morbidade estratificado por sexo: (A) mulheres e (B) homens. Brasil, 2019.

MM: multimorbidade.

A Tabela 2 apresenta as razões de prevalências brutas e ajustadas para o absenteísmo, carga de morbidade e de multimorbidade, para a população geral e estratificado por sexo. Na população em geral, ter multimorbidade aumentou em 2,6 vezes o relato de absenteísmo no trabalho em comparação com indivíduos sem multimorbidade. Após ajuste progressivo dos modelos por fatores sociodemográficos e de saúde, houve uma redução na força de associação. Em geral, ter multimorbidade aumentou em 38% (IC95% 1,04–1,82) o relato de absenteísmo. Em relação à carga de morbidade, mesmo após ajuste progressivo, o aumento do número de morbidades aumentou o relato de absenteísmo, sendo 60% maior (IC95% 1,02–2,52) entre aqueles com duas morbidades e 2,23 vezes maior (IC95% 1,49–3,35) entre aqueles com seis morbidades ou mais (Tabela 2).

**Tabela 2:** Razão de prevalência (RP) da associação entre multimorbidade e carga de morbidade com o absenteísmo bruta e ajustada, geral e estratificada por sexo.

Variáveis		Geral			Mulheres			Homens		
		Modelo 1 RP (IC95%)	Modelo 2 RP (IC95%)	Modelo 3 RP (IC95%)	Modelo 1 RP (IC95%)	Modelo 2 RP (IC95%)	Modelo 3 RP (IC95%)	Modelo 1 RP (IC95%)	Modelo 2 RP (IC95%)	Modelo 3 RP (IC95%)
Multimorbidade		2,60 (1,96–3,45)	1,72 (1,32–2,26)	1,38 (1,04–1,82)	4,11 (2,45–6,88)	2,52 (1,49–4,26)	1,73 (1,01–2,96)	1,94 (1,39–2,70)	1,43 (1,10–1,87)	1,27 (0,96–1,71)
Carga de morbidade										
	1	1,87 (1,17–2,96)	1,64 (1,08–2,50)	1,49 (0,99–2,24)	4,71 (2,10–10,58)	4,00 (1,85–8,61)	3,14 (1,50–6,58)	1,25 (0,77–2,03)	1,18 (0,76–1,74)	1,16 (0,75–1,79)
	2	3,02 (1,94–4,70)	2,05 (1,34–3,15)	1,60 (1,02–2,52)	8,36 (4,09–17,08)	5,6 (2,69–11,71)	3,54 (1,62–7,78)	1,91 (1,17–3,11)	1,45 (0,95–2,21)	1,29 (0,80–2,08)
	3	4,10 (2,72–6,16)	2,60 (1,70–3,97)	2,00 (1,31–3,05)	10,0 (4,94–20,23)	7,05 (3,25–15,29)	4,56 (2,21–9,43)	2,57 (1,67–3,97)	1,74 (1,18–2,56)	1,52 (1,01–2,28)
	4	2,70 (1,54–4,74)	2,01 (1,22–3,30)	1,28 (0,78–2,12)	9,32 (4,19–20,74)	6,28 (2,80–14,06)	2,46 (1,02–5,96)	1,36 (0,61–3,06)	1,17 (0,64–2,13)	1,11 (0,62–1,97)
	5	4,92 (3,23–7,50)	2,59 (1,56–4,29)	2,13 (1,30–3,49)	16,95 (8,66–33,16)	7,4 (3,43–16,03)	6,25 (3,06–12,76)	2,48 (1,54–4,00)	1,96 (1,00–3,82)	1,53 (0,81–2,91)
	6+	5,61 (3,8–8,26)	3,04 (1,98–4,66)	2,23 (1,49–3,35)	19,07 (10,13–35,88)	9,17 (4,57–18,39)	4,76 (2,33–9,77)	2,94 (1,94–4,44)	1,67 (1,17–2,40)	1,58 (1,11–2,26)

IC95%: intervalo de confiança a 95%. Modelo 1: modelo bruto; Modelo 2: idade, escolaridade e raça/cor; Modelo 3: modelo 2 + autoavaliação de saúde.

Na análise estratificada por sexo, ter multimorbidade aumentou 73% (IC95% 1,01–2,96) o relato de absenteísmo na população feminina, entre os homens não houve associação após ajuste progressivo por fatores sociodemográficos e de saúde [RP 1,27 (IC95% 0,96–1,71)] (Tabela 2). Entre as mulheres, a carga de morbidade apresentou uma forte associação com o relato de absenteísmo, sendo três vezes maior (IC95% 1,50–6,58) para aquelas com uma morbidade e 6,3 vezes (IC95% 3,06–12,76) entre as mulheres com seis ou mais morbidades (Tabela 2). Para o sexo masculino, após ajuste progressivo, a associação entre carga de morbidade e o absenteísmo apresentou significância estatística apenas entre aqueles com seis morbidades ou mais, sendo 58% (IC95% 1,11–2,26) maior o relato de absenteísmo entre eles, em comparação aqueles sem nenhuma morbidade (Tabela 2).

## DISCUSSÃO

Os resultados deste estudo evidenciaram a magnitude das DCNT entre indivíduos em condição de ocupação no Brasil, onde cerca de 50% possuem ao menos uma DCNT avaliada. Além disso, o padrão de morbidades mais prevalentes entre homens e mulheres que relataram absenteísmo mostrou-se diferente. Um quarto da população tem multimorbidade e a sua prevalência entre as mulheres que relataram absenteísmo foi substancialmente mais alta em comparação aos homens. Encontramos associações significativas entre o número de condições crônicas, multimorbidade e o absenteísmo no trabalho, sendo mais importante essa relação entre as mulheres.

Estima-se que mais de 43 milhões de brasileiros ocupados tenham pelo menos uma DCNT avaliada e cerca de 850 mil relataram ter faltado ao trabalho em 2019. O resultado de um estudo europeu aponta para um aumento de 3,6 para 5,2 milhões de pessoas que faltaram ao trabalho por doença entre 2006 e 2020^15^. A prevalência de absenteísmo foi de 38,5% em nossa amostra. Estudos realizados no Canadá e nos Estados Unidos, em 2016, encontraram uma prevalência de absenteísmo de 27,8% e 53% entre trabalhadores, respectivamente^16,17^.

Quanto a prevalência do número de condições crônicas em nossa população de estudo, Zhang et al. encontraram prevalências semelhantes às nossas para carga de morbidades, com aproximadamente 45% da população com pelo menos uma doença crônica^16^. Já para multimorbidade, nosso estudo encontrou uma prevalência de 23,6%, valor maior que o encontrado em outro estudo que avaliou trabalhadores em idades mais avançadas^18^. Por outro lado, a prevalência encontrada em nosso estudo é quase a metade quando comparada a um estudo realizado com trabalhadores na Austrália (2020), que encontrou uma prevalência de multimorbidade de 53,1% para a população total, 63,5% para mulheres e 41,5% para homens^8^. Cabe destacar que as diferenças encontradas na prevalência de DCNT em nosso estudo e nas pesquisas realizadas em países da Europa, podem ser explicadas, em parte, pela transição epidemiológica destes países, que estão em estágios mais avançados, representando a maior causa de morbimortalidade dessa população, em comparação com o Brasil^19,20^.

As doenças mais prevalentes em ambos os sexos, entre aqueles que relataram absenteísmo, foram problemas crônicos de coluna, hipertensão arterial sistêmica e dislipidemia. Alguns estudos também encontraram como condições crônicas mais prevalentes: doenças do sistema musculoesquelético, transtornos mentais e comportamentais, doenças do sistema circulatório, enxaqueca, colesterol elevado, doenças do aparelho respiratório e doenças do aparelho digestivo^3,8,16,17^.

Todavia, aqueles que avaliaram as diferenças entre os sexos, encontram diferenças nos diagnósticos mais prevalentes entre eles, assim como o encontrado na presente pesquisa. No estudo de Timp et al., os diagnósticos mais prevalentes para as mulheres foram os transtornos mentais, enquanto para os homens as doenças do sistema musculoesquelético foram mais prevalentes^3^.

No que se refere às ausências por razões de saúde entre o público feminino, observa-se uma tendência ao afastamento mais frequente devido a distúrbios mentais e problemas musculoesqueléticos, como os problemas crônicos de coluna e doenças do aparelho circulatório. Esses padrões podem ser reflexos dos múltiplos papéis sociais desempenhados pelas mulheres e da segregação de gênero no mercado de trabalho, sendo esse grupo mais submetido a ocupações caracterizadas por demandas físicas e emocionais elevadas, baixos salários, apoio social limitado e perspectivas de carreira pouco promissoras, aumentando o risco de licenças médicas^10^.

Além disso, as mulheres são predominantemente empregadas nos setores de saúde, serviços sociais e educação, enquanto os homens são mais comumente encontrados em empregos nos setores de construção, manufatura, tecnologia da informação e transporte^21^. Alguns estudos sugerem que maiores taxas de absenteísmo por doença estão associadas a ocupações dominadas por mulheres. A natureza emocionalmente exigente destes empregos geralmente envolve trabalhar diretamente com pacientes ou clientes, exigindo uma recuperação mais completa de transtornos mentais antes que o trabalho possa ser retomado, por exemplo^21,22^.

Ademais, a desigualdade de sexo no absenteísmo fica evidente ao analisar os resultados do estudo de Almeida e Fernandes, em que a força de trabalho é predominantemente masculina, mas as ausências por motivos de saúde são mais frequentes entre as mulheres. Sendo as doenças osteomusculares, como problemas na coluna, na região lombar e nos ombros, responsáveis pelo maior número de licenças médicas e dias perdidos, correlacionado a níveis mais baixos de escolaridade e funções ocupadas pelas mulheres na empresa^23^.

A prevalência de absenteísmo entre mulheres reduziu a partir de três condições e se manteve estável nas categorias seguintes. Para os homens, esse padrão ocorre a partir de quatro condições. Um estudo de coorte australiano sobre o absenteísmo entre jovens trabalhadores destaca a importância da sinergia entre doenças, sugerindo um efeito parcialmente aditivo, em vez de multiplicativo, na relação entre o absenteísmo e mais condições^8^. Segundo os autores, é provável que os efeitos sinérgicos de várias combinações de doenças variem desde a amplificação do efeito de condições individuais até a melhoria do efeito. Não obstante, ressaltam que a análise destes efeitos sinérgicos merece uma investigação mais aprofundada para compreender as combinações de doenças que podem ser particularmente prejudiciais^8^.

Outrossim, os resultados deste estudo apontam para uma desigualdade segundo sexo na relação entre carga de morbidade, multimorbidade e prevalência de absenteísmo. Um estudo realizado em 2021 analisou as causas de absenteísmo em 32 países da Europa e encontrou diferenças nas taxas de absenteísmo entre homens e mulheres. A diferença foi mais ampla na França, seguida do Reino Unido, Espanha e Polônia respectivamente. Nesses países, a mesma tendência foi observada: há maiores taxas de ausência por doença em mulheres que em homens^15^.

Bekker et al. realizaram uma revisão da literatura sobre a relação entre gênero e ausências por doença, descobrindo que as mulheres faltam geralmente com mais frequência, especialmente quando se trata de ausências de curta duração^24^. Além disso, encontraram que as diferenças de gênero nas faltas por doença são influenciadas por vários fatores, como o país de residência, a idade e o grupo profissional^24^.

Diferenças nas atividades diárias e nas características ocupacionais também podem influenciar a frequência e a duração das ausências por doença. As mulheres tendem, geralmente, a dedicar mais tempo às tarefas domésticas e aos cuidados dos filhos do que os homens. A hipótese da dupla carga sugere que a combinação de diferentes papéis, como os cuidados com os filhos e a jornada de trabalho externa, podem aumentar o estresse e, consequentemente, aumentar o risco de ausência por doença^3,25,26^.

Por fim, ter multimorbidade esteve associado ao relato de absenteísmo no trabalho, sendo mais importante entre as mulheres. Apesar das diferenças na prevalência de multimorbidade segundo sexo, na população em geral, Troelstra et al. encontrou associação entre a multimorbidade e a perda de produtividade no trabalho, mesmo quando ajustada pela ocupação^8^. Outro estudo também demonstrou que pessoas com a multimorbidade apresentaram taxas mais elevadas de absenteísmo e presenteísmo do que qualquer doença crônica isolada ou nenhuma doença crônica^9^.

Os trabalhadores que apresentam multimorbidade ocupam uma parcela considerável de gastos pessoais e do Sistema Único de Saúde (SUS) devido às suas doenças. No Brasil, em 2013, foram realizadas 974.641 internações hospitalares por DCNT, totalizando um custo de R$ 1.848.627.410,03 (US$695,6 milhões) para o SUS^27^. Uma alternativa para redução do impacto das DCNT na saúde dos trabalhadores está no entendimento de que o local de trabalho exerce uma influência direta sobre a saúde e estilo de vida deles, visto que, nesse ambiente, passam grande parte do seu tempo^28^.

Ao identificar as doenças mais comuns que levam ao absenteísmo é possível desenvolver políticas de saúde ocupacional focadas na prevenção e no tratamento dessas condições. Isso pode incluir programas de vacinação, campanhas de conscientização, exames periódicos, ergonomia no local de trabalho e suporte emocional.

Mais ainda, compreender a magnitude e impacto das DCNT pode ajudar no desenvolvimento de políticas de trabalho mais flexíveis, como o *home office* ou jornadas reduzidas. Essas alternativas podem permitir que os trabalhadores continuem contribuindo sem a necessidade de afastamentos prolongados. Além disso, o apoio à saúde mental e física dos trabalhadores cria um ambiente mais saudável e colaborativo.

Os pontos fortes do presente estudo incluem o uso de dados com representatividade nacional. O uso de dados com caráter periódico, como a PNS, permite análises futuras quanto ao comportamento e acompanhamento da população de estudo. As limitações do estudo compreendem o uso de medidas autorreferidas de morbidade e absenteísmo que podem subestimar sua prevalência. Além disso, todas as doenças avaliadas foram consideradas igualmente, embora o efeito da carga de morbidade e de multimorbidade possa variar com a combinação e a gravidade das DCNT^14^.

Importante salientar que o conjunto das quatorze condições crônicas avaliadas podem ter subestimado a estimativa de prevalência de carga de morbidade e de multimorbidade na população de estudo. Ainda, a variabilidade no número e na lista de doenças incluídas em estudos anteriores dificultam a comparação da prevalência e seu impacto sobre o absenteísmo. Outra limitação importante é que o absenteísmo foi avaliado como a ausência (sim/não) do trabalho, sem considerar o tempo de afastamento. Deste modo, os resultados devem ser interpretados considerando que a relação entre as DCNT e perda de produtividade pode ser diferente da encontrada pelo relato medido de forma dicotômica. Além disso, por se tratar de um estudo transversal, as associações aqui encontradas não avaliaram a causalidade, considerando que a exposição e o desfecho foram avaliados simultaneamente.

Em conclusão, o presente estudo concluiu que as DCNT já são altamente prevalentes entre os trabalhadores brasileiros e que a carga de morbidades e de multimorbidade está fortemente relacionada com o absenteísmo no trabalho, especialmente entre as mulheres. Considerando que os aumentos das DCNT nos próximos anos e da razão de dependência, com uma população mais envelhecida, coloque um fardo maior sobre os jovens trabalhadores, combater os efeitos das DCNT nessa população é essencial, para os seus empregadores e para a sociedade em geral.

Por fim, estes resultados destacam a importância da prevenção e da gestão precoce destinada a minimizar o impacto das condições crônicas não transmissíveis entre os trabalhadores. Dentre as ações visadas estão: as estratégias de redução da multimorbidade, o aumento da capacidade dos trabalhadores para lidar com suas condições e a melhoria do acesso aos serviços de saúde para prevenção e tratamento para os trabalhadores com doenças, especialmente para as mulheres.
